# Immuno Nanosensor for the Ultrasensitive Naked Eye Detection of Tuberculosis

**DOI:** 10.3390/s18061932

**Published:** 2018-06-14

**Authors:** Noremylia Mohd Bakhori, Nor Azah Yusof, Jaafar Abdullah, Helmi Wasoh, Siti Suraiya Md Noor, Nurul Hanun Ahmad Raston, Faruq Mohammad

**Affiliations:** 1 Institute of Advanced Technology, Universiti Putra Malaysia, 43400 UPM Serdang, Malaysia; noremyliamb@gmail.com; 2 Department of Chemistry, Faculty of Science, Universiti Putra Malaysia, 43400 UPM Serdang, Malaysia; jafar@upm.edu.my; 3 Faculty of Biotechnology and Biomolecule Science, Universiti Putra Malaysia, 43400 UPM Serdang, Malaysia; helmi_wmi@upm.edu.my; 4 School of Medical Sciences, Universiti Sains Malaysia, Kubang Kerian, 16150 Kelantan, Malaysia; ssuraiya@usm.my; 5 School of Biosciences and Biotechnology, Faculty of Science and Technology, Universiti Kebangsaan, Malaysia, 43600 UKM Bangi, Malaysia; nurulhanun@ukm.edu.my; 6 Surfactant Research Chair, Department of Chemistry, College of Science, King Saud University, P.O. Box 2455, Riyadh 11451, Saudi Arabia

**Keywords:** tuberculosis, immune nanosensor, plasmonic ELISA, biosensors, naked eye detection

## Abstract

In the present study, a beneficial approach for the ultrasensitive and affordable naked eye detection and diagnosis of tuberculosis (TB) by utilizing plasmonic enzyme-linked immunosorbent assay (ELISA) via antibody-antigen interaction was studied. Here, the biocatalytic cycle of the intracellular enzymes links to the formation and successive growth of the gold nanoparticles (GNPs) for ultrasensitive detection. The formation of different colored solutions by the plasmonic nanoparticles in the presence of enzyme labels links directly to the existence or non-existence of the TB analytes in the sample solutions. For disease detection, the adapted protocol is based mainly on the conventional ELISA procedure that involves catalase-labeled antibodies, i.e., the enzymes consume hydrogen peroxide and further produce GNPs with the addition of gold (III) chloride. The amount of hydrogen peroxide remaining in the solution determines whether the GNPs solution is to be formed in the color blue or the color red, as it serves as a confirmation for the naked eye detection of TB analytes. However, the conventional ELISA method only shows tonal colors that need a high concentration of analyte to achieve high confidence levels for naked eye detection. Also, in this research, we proposed the incorporation of protein biomarker, Mycobacterium tuberculosis ESAT-6-like protein esxB (CFP-10), as a means of TB detection using plasmonic ELISA. With the use of this technique, the CFP-10 detection limit can be lowered to 0.01 µg/mL by the naked eye. Further, our developed technique was successfully tested and confirmed with sputum samples from patients diagnosed with positive TB, thereby providing enough evidence for the utilization of our technique in the early diagnosis of TB disease.

## 1. Introduction

*Mycobacterium tuberculosis* (*M. tuberculosis*) is an obligate pathogenic bacterium that is responsible for the life threatening infectious disease that occurs worldwide called tuberculosis (TB) [1]. Critical health issues have arisen from TB infection since the disease is easily spread through the respiratory tract [2] and there has been an alarming increase in TB resistance to the growing number of drugs [3]. Accordingly, early detection of TB disease is crucial, however, this remains challenging due to the lack of accurate and rapid techniques, clinical laboratories and skilled personnel for efficient diagnosis and treatment of the disease.

Recently, various TB marker proteins have been employed for the diagnosis and treatment of TB and related diseases [4,5]. One of these is the ESAT-6-like protein EsxB or CFP-10, which is a 10 kDa secreted antigen from *M. tuberculosis* that has been identified as an excellent candidate for use as a TB biomarker for early disease detection. Hence, CFP-10 has been selected as an alternative marker for the development of new sensing methods. This protein is an early secretory antigen and offers the strongest interaction with its cognate antibody when compared with other *M. tuberculosis* antigens. It is also found abundantly in the culture filtrate of *M. tuberculosis* and is not present in *M. bovis* BCG and most other non-tuberculosis *Mycobacteria* strains [5].

Traditional microbial culture-based tests are typically used to detect TB in clinical practice [6]. However, these conventional techniques are laborious and time consuming due to the slow-growing type *M. tuberculosis*. In order to improve TB detection, various biosensing techniques such as nucleic acid amplification tests [7,8,9], latex agglutination [10], enzyme-linked immunosorbent assay (ELISA) [11], radiometric detection [12], and flow cytometry [13] have been implemented. These techniques provide rapid and improved sensitivity and specificity functions for diagnosis of TB but many have limitations with regard to providing real-time detection results and also require highly skilled personnel to handle the lengthy procedures and complex instrumentation. For example, the use of sputum smear microscopy (conventional microscopy) for the diagnosis of pulmonary TB disease suffers from the major drawback of low sensitivity, especially for patients co-infected with HIV. As an alternative, fluorescence microscopy provides high accuracy, sensitivity, specificity and incremental yield, although this technique is limited by the high cost and the need for programmed methods/conditions with trained personnel [9]. Similarly, nucleic acid amplification tests are frequently used in the United States and have been commercially available for the last two decades and the accuracy and specificity of this test is far better than the sputum smear and fluorescence microscopies. The major limiting factor of this test is the high cost associated with the diagnosis as well as the requirement for respiratory specimens from the patients for the analysis, when the collection of such samples from patients may not be possible in all cases. Moreover, the decisions about the management of TB cases are based on the test results as this plays a vital role in the confirmation of the disease and commencement of the treatment process, i.e., a method that promotes the early initiation of treatment is vital [7,8]. The latex agglutination test uses tuberculoprotein as an antigen and so in most cases the test is simple, accurate, rapid and can be easily used in remote areas; however, the limiting factors of this test is the stability of antigen-coated beads and other testing reagents as they are time bound, and the time scale required for the total sample analysis [10]. Other tests such as ELISA, flow cytometry, etc. are available, but they too suffer from limitations associated with high costs, complex assay handling, the need for expensive instruments and trained personnel, and the difficulties of transferring the test to remote rural regions, especially in developing nations [11,12,13]. In order to efficiently diagnose TB, the test should be widely available, user-friendly, require minimal or no-training of personnel and produce highly accurate outcomes which are available for use in areas with peripheral resources.

In our approach, we adapted plasmonic ELISA to develop ultrasensitive direct naked eye detection of TB with a high confidence level for point-of-care (POC) testing, which does not require trained personnel and is portable, thus, it can be applied in resource-constrained countries. In this technique, the biocatalytic cycle of the intracellular enzymes links to the formation and successive growth of the gold nanoparticles (GNPs) for ultrasensitive detection. The formation of different colored solutions (blue or red) by the plasmonic nanoparticles in the presence of enzyme labels confirms directly the existence or non-existence of the TB analytes in the sample solutions. Although lateral flow tests (LFTs) are more popular when it comes to being low-cost, easy, rapid and portable detection of TB, [14,15], we believe that the plasmonic ELISA method is more sensitive than the corresponding LFT method as it enables the naked eye detection of various disease biomarkers down to attomolar concentration [16]. Theoretically, any particle that gives color can be used for the LFTs, such as latex which gives a blue color and similarly the GNPs, as they are responsible for the development of red- or blue-colored solutions easily. Since the GNPs are known to be the most popular agents used in the colorimetric assays, and the application of these particles in recent years has been extended to the plasmonic ELISA too because of its high sensitivity and responsible color contrasting behavior that can be highly applicable to detection probes [17,18,19].

## 2. Materials and Methods

### 2.1. Chemicals

Streptavidin, catalase from bovine liver, albumin from bovine serum (BSA), gold(III) chloride trihydrate, 2-(*N*-morpholino) ethanesulfonic acid (MES), Tween-20 (T-20), hydrogen peroxide (H_2_O_2_), sulfuric acid (H_2_SO_4_), l-Gluthathione reduced (LGT), dimethyl sulfoxide (DMSO) were purchased from Sigma-Aldrich (Kuala Lumpur, Malaysia). Phosphate buffer saline (PBS) tablets were purchased from Amresco (South Logan, UT, USA) and 24-unit ethylene glycol functionalized (SM(PEG)24) with succinimidyl and maleimido ends were purchased from Thermo Fisher Scientific (Waltham, MA, USA). 3,3′,5,5′-Tetramethylbenzidine (TMB) ELISA substrate (highest sensitivity), *Mycobacterium tuberculosis* ESAT-6-like protein esxB (CFP-10), mouse monoclonal anti CFP-10, biotin-labelled goat polyclonal secondary antibody to mouse and horseradish peroxidase (HRP)-labelled goat polyclonal secondary antibody to mouse were purchased from Abcam (Cambridge, UK). PD-10 disposable column was from GE Healthcare Life Sciences (Chemopharm Sdn Bhd, Petaling Jaya, Selangor, Malaysia) and 96-well polystyrene disposable ELISA plates were used (Mid Western, Kuala Lumpur, Malaysia).

### 2.2. Conjugation of Streptavidin with Catalase

For the conjugation, 1 mL of streptavidin (1 mg/mL in PBS) was mixed with 4 μL of SM(PEG)24 (250 mM in dry DMSO) and incubated at room temperature (25 °C) for 30 min. The desalting column was used for the removal of excess cross linker. Then, 5 mg of catalase was added and the solution was kept at 4 °C overnight. Finally, the streptavidin-catalase conjugate was aliquoted and stored at 4 °C until its further use.

### 2.3. ELISA

For the detection of CFP-10, we first coated the ELISA plate with 100 μL of CFP-10 in carbonate buffer and then incubated it for 1.5 h. Following this, the plate was washed three times with PBS pH 7.6 and 0.05% Tween-20 (PBST) by tapping it against a clean paper towel. Then, the plate was blocked with 370 μL of PBS containing BSA (PBSA) (1 mg/mL) for 1.5 h. All the antibodies and enzyme conjugates were diluted in diluent antibody containing PBST and 1% BSA. The plate was washed three times with PBST, and the plate (invert) was kept at 4 °C for 2 h. Then, 100 μL of monoclonal anti CFP-10 antibody as the primary antibody was added to the plate at 4 °C for 1.5 h. After 1.5 h, the plate was washed three times with PBST and 100 μL of biotinylated polyclonal secondary antibody was added to the plate and incubated for another 1.5 h at 4 °C. The plate was then washed three times and 100 μL of catalase-streptavidin conjugate (*v*/*v* 1:20) was pipetted into the plates and left for 1.5 h at 4 °C. After this period, the wells in the plate were washed three times with PBST, two times with PBS, one time with deionized water and then dried. Then, 100 μL of hydrogen peroxide (in 1 mM MES, pH 6.5) buffer was pipetted into the wells. Immediately, 100 μL of gold ion solution freshly prepared in 1 mM MES buffer was added to the wells at room temperature. At this stage, the GNPs formation in the form of colored solution could be seen and read with a microplate reader at an absorbance of 550 nm. For the analysis of real samples, the sputum from positive and negative TB patients were diluted in 4% sodium hydroxide first, and then proceeded to the same coating process as mentioned above.

### 2.4. Instrumental Analysis

For the absorbance study, we applied a UV-Vis spectrophotometer from PerkinElmer (Waltham, MA, USA) and a Benchmark microplate reader (Bio-Rad Laboratories, Inc., International Business Park, Singapore). For the transmission electron microscopic (TEM) studies, JEM-2100F Transmission Electron Microscope (Peabody, Saint Louis, MO, USA) was used.

## 3. Results and Discussion

**Figure 1:**
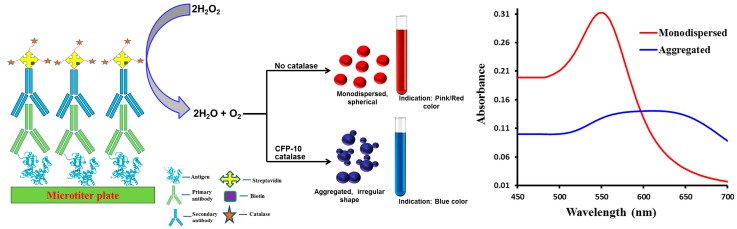
Schematic representation of the principle involved for the naked eye TB detection and the corresponding absorption spectra for the monodispersed and aggregated particles. With the use of this technique, the formation of pink/red colored solution means that the GNPs are monodispersed, no catalytic reaction and so no *M. tuberculosis*. Similarly, formation of blue colored solution indicates the formation of aggregated GNPs, and a CFP-10 mediated catalytic reaction which supports the presence of *M. tuberculosis*.

In this study, an immuno nanosensor was developed to detect TB protein marker, CFP-10. The ELISA format for both conventional and plasmonic types was adopted in this experiment. Briefly, for both conventional and plasmonic ELISAs, a target molecule was coated on the disposable 96-well plate. The target was then captured by the target-specific primary antibody. Subsequently, the secondary antibody was linked with an enzyme via biotin-streptavidin binding. The enzymatic reaction of substrate produced a color solution and the intensity of the colored product can be read by microplate reader. In conventional ELISA, the HRP catalyzed the conversion of colorless TMB solution into blue in the presence of target, and then changed to yellow with the addition of 2 M of H_2_SO_4_ as a stop solution. The absence of target produced colorless solution in this format. The plasmonic ELISA utilizes the enzymatic biocatalysis of catalase in the presence of hydrogen peroxide that is linked to the growth of GNPs, which produced red or blue colored solutions in the absence or presence of target, respectively. The formation of GNPs with different morphological and optical properties is schematically represented in Figure 1, in which the plasmonic ELISA links the formation of colored solution by the nanoparticles to the presence or absence of TB analyte. The technique is based on the enzyme’s catalytic cycle connected to the formation GNPs for ultrasensitive naked eye detection. The formation these GNPs with the addition of gold(III) chloride in either blue or red color confirms whether TB analytes are available or not in the sample solutions. Since the conventional ELISA assay generates colorless solution in the absence of target, and some light-colored solution in the presence of a low concentration of target, thereby leading to biased results which further limit its application to real-time sample analysis. Furthermore, in conventional ELISA, naked eye detection can only be achieved when a high concentration of target molecule is present and thus, it is not suitable for ultrasensitive detection by the naked eye.

**Figure 2:**
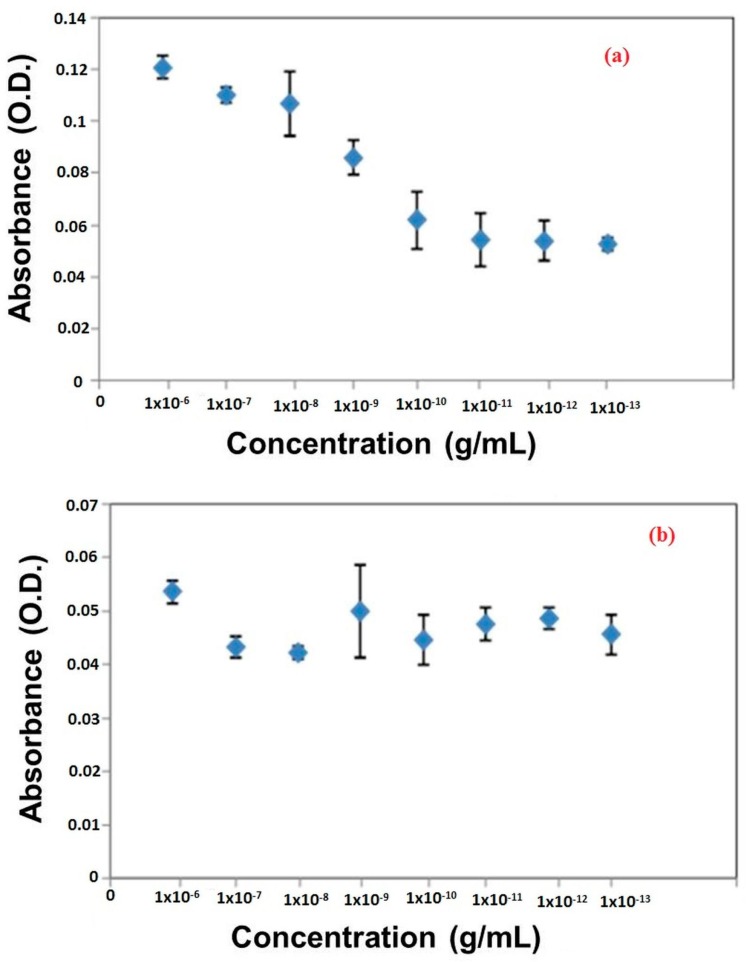
Optimization of (**a**) primary and (**b**) secondary antibodies.

With the application of a plasmonic ELISA platform, the sensitivity of target detection can be improved even at the level of the naked eye. For that, firstly, successive binding of CFP-10 with primary antibodies and further binding of secondary antibodies were confirmed using the conventional ELISA method. As shown in Figure 2, the concentration of antibodies was optimized to get the highest detection of CFP-10. According to the results, the optimum concentrations of primary and secondary antibodies were 1 × 10^−10^ g/mL and 1 × 10^−7^ g/mL, respectively. Therefore, the formation of the antigen-antibody complex was successful as the signal generation read at an absorbance of 550 nm.

**Figure 3:**
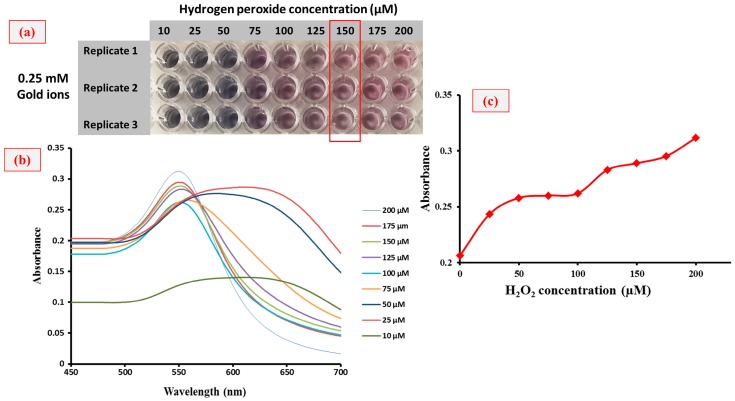
(**a**) Reduction of gold ion to GNPs, (**b**) absorption spectra for the peak at 550 nm, and (**c**) the absorbance curve towards the formation of GNPs at different concentrations (10–200 µM) of hydrogen peroxide.

**Figure 4:**
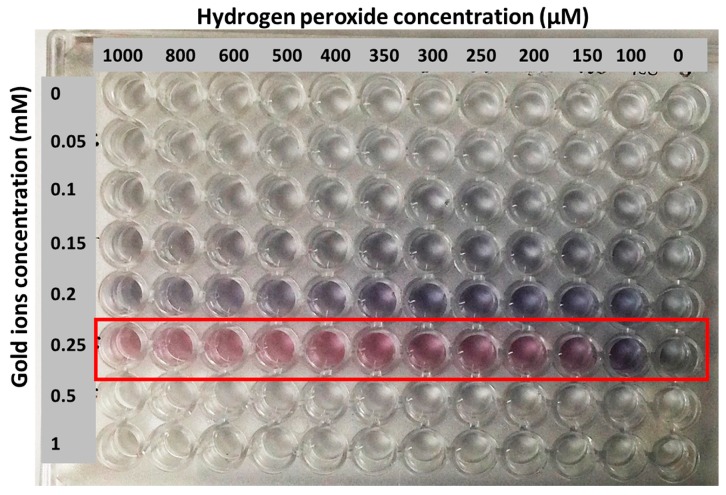
Generation of nanoparticle solutions with different colors depending on the concentration of hydrogen peroxide and gold ions.

Figure 3a–c shows the reduction reaction of gold ions which generated color changes in the solution along with the absorbance spectra at different concentrations. The extremely sensitive hydrogen peroxide made it possible to use as a reducing agent in this reaction. Gold ion was reduced by hydrogen peroxide in MES buffer to obtain GNPs. In this approach, the GNPs’ color formation was affected by the concentration of hydrogen peroxide present in the reaction mixture (Figure 3b), which produces either blue- or red-colored solutions. Accordingly, catalase was used to consume the hydrogen peroxide. Therefore, in the presence of target (CFP-10), hydrogen peroxide was consumed by the catalase and its concentration decreased; this is responsible for the slow reduction rate of gold ions into GNPs. In this case, the occurrence of GNPs agglomeration produces blue-colored solution. However, in the absence of target and associated catalase enzymes, high amounts of hydrogen peroxide cause the rapid kinetic growth of GNPs, which results in the generation of non-aggregated GNPs to form a red-colored solution. Most importantly, this plasmonic ELISA platform is appropriate for naked eye detection, because the tonality of the colored solution produced could be easily distinguished, even at a simple glance. To this concern, the effect of hydrogen peroxide concentration on the growth of GNPs was studied in order to set an optimal negative control condition and the results are shown in Figure 4. A wide range of hydrogen peroxide and gold ion concentrations were tested. From the results, the gold ions at 0.25 mM were chosen as the optimum concentration, and this value was fixed for further experiments. Next, the concentration of hydrogen peroxide was optimized in a range of 10 to 200 µM as shown in Figure 3a–c. It can be seen in Figure 3a that the color tonality of solutions changes from blue to red with an increase in the concentration of hydrogen peroxide. Here, the optimum concentration of hydrogen peroxide at 150 µM was chosen for plasmonic ELISA because this is the lowest concentration of hydrogen peroxide used to give a significant color change. Based on the effect of different concentrations of hydrogen peroxide on the absorbance of GNPs formed, the concentration of 150 µM for hydrogen peroxide is stable and good enough as a negative control.

**Figure 5:**
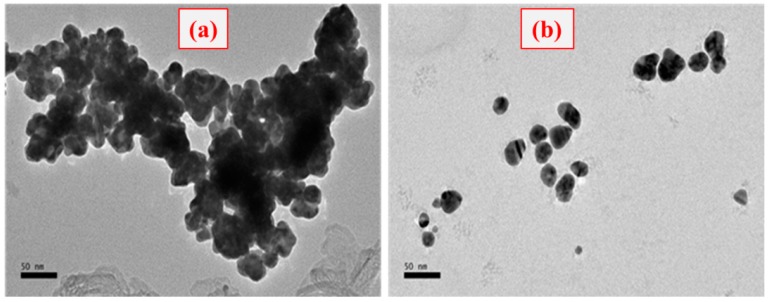
TEM images of GNPs morphology and state of aggregation of GNPs grown according to the reaction, (**a**) in the presence of CFP-10 and (**b**) in the absence of CFP-10.

The morphology of GNPs cluster formation was also confirmed by the TEM analysis. As shown in Figure 5, the agglomeration of GNPs seems to be occurred when low amounts of hydrogen peroxide are present, thus causing the nanoparticle cluster (fully agglomerated) containing solution to be blue colored (Figure 5a). In contrast, the solution containing distributed GNPs (monodispersed) was red colored when a high amount of hydrogen peroxide is present (Figure 5b).

**Figure 6:**
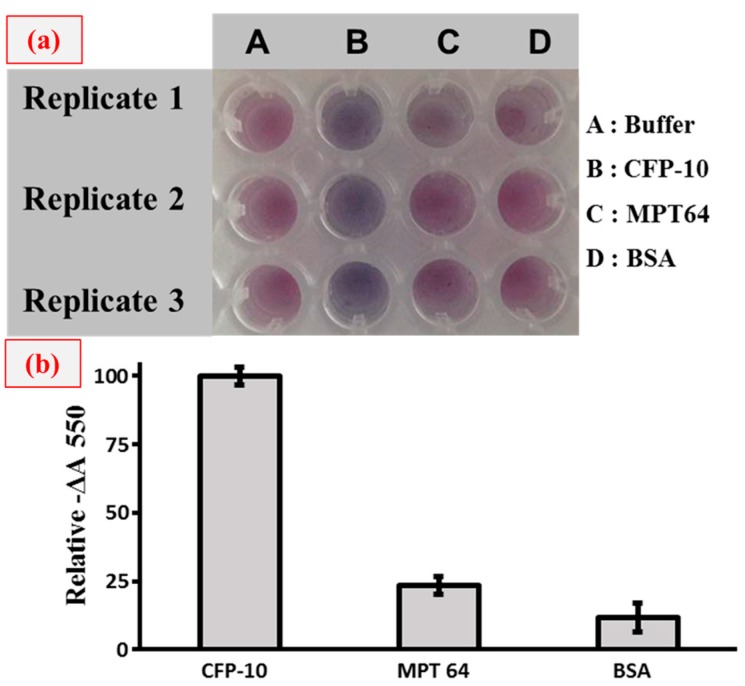
Specificity test of plasmonic ELISA analysis for the CFP-10 detection. (**a**) Naked-eye detection using plasmonic ELISA and (**b**) the relative signal (−∆A 550) is expressed as the decrease in absorbance with respect to the blank monitored at 550 nm, corresponding to the CFP-10 target signal response. The plasmonic ELISA was tested to observe the specific binding to CFP-10 and other protein targets (MPT 64 and BSA). For the study, a concentration of 50 ng/mL was applied for each sample.

Following this, specificity testing was carried out to validate whether this plasmonic ELISA platform could be used to specifically detect CFP-10 protein markers. Figure 6 shows the specificity analysis when we applied another TB protein marker, MPT64 and also the BSA complex protein. As a result, the blue colored solution can only be seen in the sample containing the CFP-10, while the colors are not seen in the other solutions. This analysis provides further support that the plasmonic ELISA platform applied in our approach is specific to the CFP-10 protein marker.

**Figure 7:**
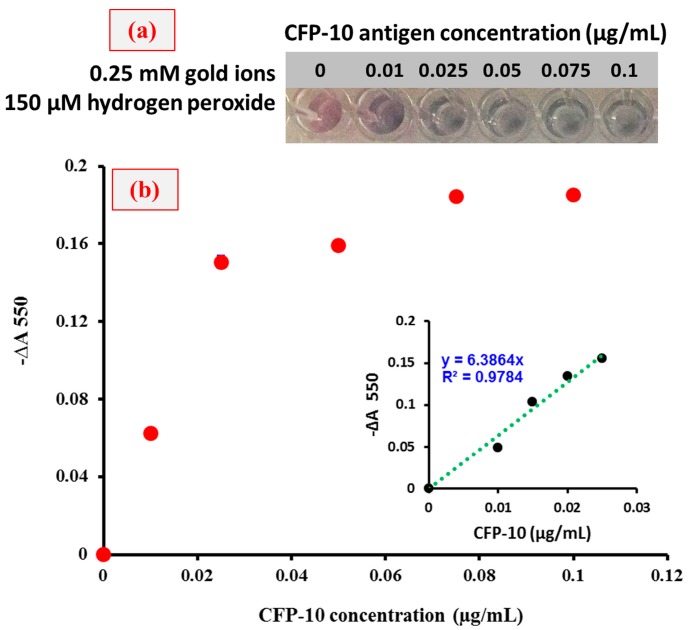
Dose-dependent binding assays by using catalase-assisted plasmonic ELISA for CFP-10 detection. (**a**) Naked-eye detection of CFP-10 using plasmonic ELISA and (**b**) the signal (−∆A 550) is expressed as the decrease in absorbance with respect to the blank monitored at 550 nm.

In order to find the minimum concentration of CFP-10 required to observe the color changes by naked eye detection, dose-dependent experiments were performed and the results are shown in Figure 7. For the analysis, CFP-10 concentrations in the range of 0–0.1 µg/mL were tested and the liner graph was plotted with an R^2^ value of 0.9784. From Figure 3 and Figure 7, the minimum concentration of CFP-10 was found to be 0.01 µg/mL so as to observe the probable changes by the naked eye and also using the spectrophotometer.

**Figure 8:**
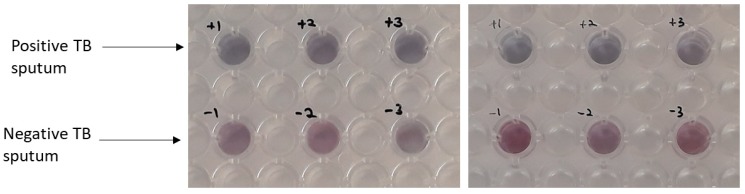
Detection of CFP-10 in sputum samples from positive TB patient and also from negative TB patient as control experiment.

Further experiments were performed in order to investigate the performance of newly developed plasmonic ELISA platform in the detection of CFP-10 in real samples. In this case, we used sputum samples from patients positively diagnosed with TB and also from negative TB patients (healthy-normal) as the control and the results are shown in Figure 8. From the analysis of data shown in Figure 8, one can clearly see the appearance of blue-colored solution only with the positive TB sputum, while no such color can be seen in the negative TB sputum samples. This result further suggests that the performance of the plasmonic ELISA platform was not disturbed by the presence of complex matrices in sputum. Therefore, the technique developed in this study could primarily be useful for the rapid analysis of real samples for the detection of TB, and thereby, contributing significantly to the clinical field.

## 4. Conclusions

In conclusion, we have demonstrated the detection of a TB biomarker, the CFP-10 protein, with both the naked eye and spectroscopy analysis by adapting a new signal generation mechanism to conventional ELISA. We believe that the developed method is easy to adopt as the technique does not require any expensive instruments or personnel and thus, can be useful for the ultrasensitive detection of CFP-10 that is linked to the diagnosis of TB infection, especially in resource-constrained countries.
